# Supplementary material for: Socio-economic urban scaling properties: Influence of regional geographic heterogeneities in Brazil
Source: PLoS One. 2020 Dec 4;15(12):e0242778. doi: 10.1371/journal.pone.0242778 (PMC7717547; doi:10.1371/journal.pone.0242778)
Supplement: S1 Appendix — (DOCX) [file pone.0242778.s001.docx]

S1 Appendix

Fig. S1: Per capita GDP of Brazil in 2016, BR$. The map was generated with GeoDa open source program.


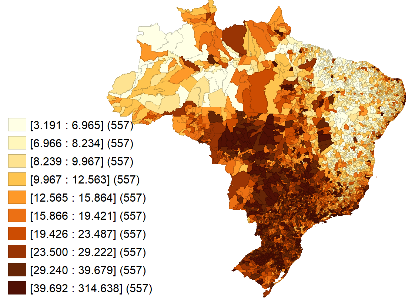


Source: Brazilian Institute of Geography and Statistics (IBGE) [33].

Figure S1 presents the spatial distribution of per capita GDP in Brazil and suggest a considerable regional heterogeneity of income in the country. This heterogeneity was tested using the statistics of Moran index of spatial autocorrelation and Local Indicators of Spatial Association (LISA), for the per capital GDP. Table S1 presents the Moran index calculated for the whole country and for the five microregions in Brazil. The spatial weight matrix used was based on binary matrix of contiguity of the neighboring municipalities of first and second order. The positive spatial autocorrelation index of 0.322 for the whole country showed that spatial distribution of per capita GDP in Brazil is heterogeneous and with a pattern of concentration of municipalities of high and low income is specific locations in the country. The intra-regional analysis showed that this heterogeneity decreases inside each region, but it still is maintained.

Table S1: Moran index of spatial autocorrelation of per capita GDP in Brazil in 2016.

| **Country/Region** | **Moran Index** |
| --- | --- |
| Brazil | 0.322 |
| Midwest | 0.195 |
| Northeast | 0.143 |
| North | 0.229 |
| Southeast | 0.184 |
| South | 0.109 |

Source: Research results.

Figure S2 presents cluster maps built from the local statistic LISA at 5% of significance for the local spatial autocorrelation among the municipalities. It is possible to verify large and statistically significant clusters of high and low per capita GDP in Brazil and inside of its five macro regions. Thus, the heterogeneity verified at the country and intra-regional level has implications for specific patterns of income concentration.

Figure S2 : Cluster maps from the local spatial autocorrelation index of per capita GDP in 2016. The maps were generated with GeoDa open source program. In the legend “High-High” means a city with high GDP with neighbors also with high GDP. The same idea may be done to interpret the other colors on the maps.

| Brazil  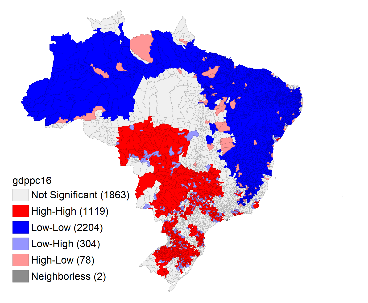 | Midwest region  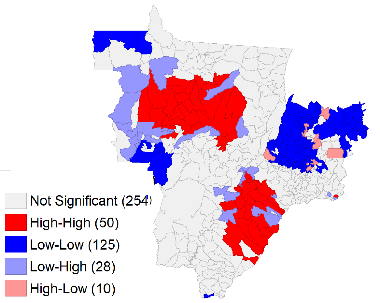 | North region  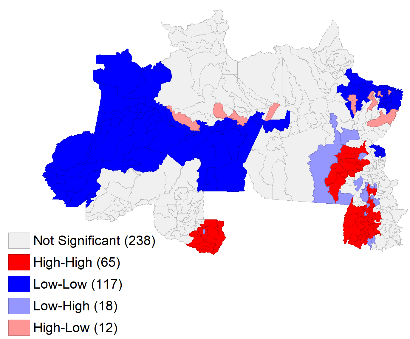 |
| --- | --- | --- |
| Northeast region  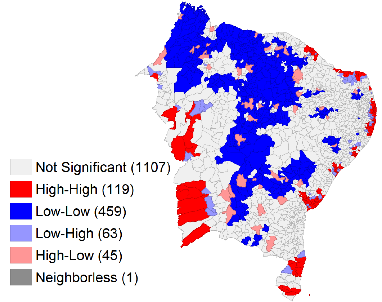 | Southeast region  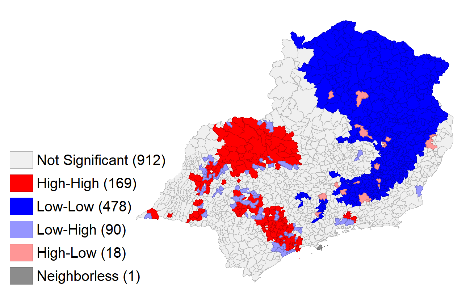 | South region  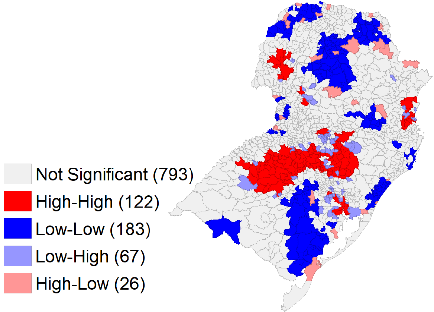 |

Source: Research results

**Methodology**

**Global Spatial Autocorrelation Moran Index.**

Considering a variable *x* at the location *i*, and the deviations from its mean, expressed as $z_{i}=(x_{i}- \bar{x})$. The Moran’s I index is the spatial autocorrelation statistic between the variable and its spatial lag:

$$I= \frac{\sum_{i} \sum_{j} w_{ij}z_{i}*z_{j}/S_{0}}{\sum_{i} z_{i}^{2}/n}$$

Where *w_ij_* are the elements of the spatial weight matrix, so that $S_{0}= \sum_{i} \sum_{j} w_{ij}$ is the sum of all the weights, and *n* as the number of observations.

**Local Spatial Autocorrelation Moran Index.**

The Local Moran statistic, also known as Local Indicator of Spatial Association (LISA), is used in general to identify local clusters and local spatial outliers. For each observation at the location *i* and the information used to calculate the Moran index, the local index is:

$$I= \frac{z_{i}\sum_{j} w_{ij}z_{j}}{\sum_{i} z_{i}^{2}}$$

The formula shows that the global index is the average of the local index. For details about both indexes and the spatial weight matrixes, see Anselin (2013)

**Reference**

ANSELIN, Luc. *Spatial econometrics: methods and models*. Springer Science & Business Media, 2013.
