# Supplementary material for: Socio-economic urban scaling properties: Influence of regional geographic heterogeneities in Brazil
Source: PLoS One. 2020 Dec 4;15(12):e0242778. doi: 10.1371/journal.pone.0242778 (PMC7717547; doi:10.1371/journal.pone.0242778)
Supplement: Attachment — Submitted filename: Response to the ReviewersPlosoOneR.docx [file pone.0242778.s002.docx]

# Response to the Reviewers

We would like to thank the editor and reviewers for their contributions. Please, find below, in red color, our point by point comments to the reviewers.

===================================================================================

Reviewer #1: Thank you for the opportunity to review this interesting paper. While I found it to be reasonably well-written and interesting, I cannot recommend publication at this time. After reading the manuscript carefully, I think the scientific contribution and novel knowledge of the research need to be specified. What problems should be solved, such as theory, method or policy of urban development in Brazil? Compared with the relevant research, where is the innovation and contribution of this study? I think some clarifications and major alterations will increment the general quality of the paper.

Author’s response: The authors thank the reviewer for reading our manuscript and for the important comments in the report. Overall, we believe that after the changes that we introduced in the new version, we have a significant improvement on our manuscript.

1.Authors need to better address the goals of the paper in the end of the Introduction section. They are poorly or insufficiently addressed.

1. As opportunely suggested by the reviewer we have included a new paragraph in the end of the introduction where we clearly address the goals of our work. Please, see text from line 62 to line 76.

2.Data sources require explanation and critical discussion.

2. We have included an additional explanation on the data in the first paragraph of the section “Results and Discussion”. Please, see text from line 124 to line 130.

3.Figure 3 and Figure 9 could be improved by drawing.

3. We have amplified Figure 9 and improved Figure 3. We hope that after the changes the figures have improved their quality.

4.Line 62: the title "Discussion" is not appropriate.

4. We thank the referee for calling our attention to this issue. We have removed “Discussion”, in line 62.

5.Further analysis on the influence of regional geographic heterogeneities is important.

5. We do agree that further analysis in order to understand completely how socio-economic heterogeneities present in continental size countries. This work intends to contribute to such discussion in Brazil, and certainly may be extended to include a larger number of urban variables. For the moment, we understand our work already uncovers a number of interesting findings. Indeed, the quantities selected for our analysis are representative of several sectors of socio-economic indicators, providing a first but wide view of their scaling properties. We intend to proceed further with our investigation, so that new insights coming from our results may be presented in a new future.

===================================================================================

Reviewer #2: Using economic, infrastructure and violence related data sets for the time interval 2002-2016 in Brazil, the study analyzed socio-economic urban scaling properties in the country. Results indicate that regional specificities related to some selected factors have a larger influence on the absolute value of the urban indexes. Regional scaling similarities and differences among regions were also uncovered. The study was good writing, and these findings are helpful for understanding the socio-economic urban scaling properties. However, there are some issues the authors must response before this manuscript to be published.

Author’s response: The authors thank the reviewer for reading our manuscript and for the important comments in the report. Overall, we believe that after the changes that we introduced in the new version, we have a significant improvement on our manuscript.

1, the study pointed that there exists regional heterogeneities in Brazil, the reviewer suggest authors test the statistical significant of the spatial stratified heterogeneities using statistic methods.

1. We have added a supporting information material which presents the Brazilian spatial heterogeneities and also the regional ones.

2, comparison of findings between the study and the other related papers should be discussed in the manuscript.

2. In the section “Conclusion” we have mentioned that our conclusions are in the same direction of the Bettencourt paper, ref. 27, which presents regional scaling analysis for India.

3, limitations and the further studies of the study should be discussed in the manuscript.

3. We have include a new paragraph in the section “conclusion”.

4, some findings have been discussed, however some of them need more scientific interpretations.

4. We believe that we have improve the discussion of our findings by including a new paragraphs in the “Introduction” and in the “Conclusion” sections.

5, the reviewer suggest authors check structure of the manuscript. For example, in line 62, “discussion” is appeared before “methods” section.

5. We thank the referee for calling our attention to this issue. We have removed “Discussion”, in line 62.
