# Supplementary material for: Socio-economic urban scaling properties: Influence of regional geographic heterogeneities in Brazil
Source: PLoS One. 2020 Dec 4;15(12):e0242778. doi: 10.1371/journal.pone.0242778 (PMC7717547; doi:10.1371/journal.pone.0242778)
Supplement: Attachment — Submitted filename: Response to the Reviewers2.docx [file pone.0242778.s003.docx]

# Response to the Reviewers

We would like to thank the editor and reviewers for their contributions. Please, find below, in red color, our point by point comments to the reviewers.

Reviewer #1: All comments have been addressed. The present document has been largely improved from its previous version.

Author’s response: The authors thank the reviewer for the previous comments. We believe that after the changes, we have an improvement in our manuscript.

Reviewer #2: This version has been improved much. Some issues, however, should be addressed. Regional geographic heterogeneities of the research object is the core content of the article, in the manuscript, the authors test the statistical significant of the spatial stratified heterogeneities using Local Spatial Autocorrelation Moran Index (LISA). The reviewer think that LISA can be used to test the local heterogeneities, however, the regional heterogeneity studied in the manuscript is more suitable to be expressed by spatial hierarchical heterogeneity, which can be calculated by geodetector q statistic (Wang, et al. A Measure of Spatial Stratified Heterogeneity. Ecological Indicators 2016), so that the reviewer suggest authors complement the analysis and added in the main text.

Author’s response: The authors thank the reviewer for this new suggestion and fully agree with it. We have included new results and a discussion of the parameter q, reported in Wang, et al. A Measure of Spatial Stratified Heterogeneity, Ecological Indicators 2016. In fact, the results we have found support our discussion regarding regional geographic heterogeneities. Metropolitan areas in every Brazilian region concentrates wealth, infrastructure, high level education employees and violence, which explain the values of q close to zero (heterogeneity within the regions are more significant than between them). The regions present similar heterogeneity, despite the discrepancy on the absolute value of the socioeconomic variables. On the other hand, the largest value of q associated with WDS indicates a comparatively smaller infrastructure discrepancy between the richest regions with the poorest ones. This can be explained, among other reasons as political and historical, by the fact that the poorer regions, as North and Northeast, have globally had less investments in infrastructure. We have included such discussion (in red) in pages 5 and 6 of the manuscript.
