# Supplementary material for: Socio-economic urban scaling properties: Influence of regional geographic heterogeneities in Brazil
Source: PLoS One. 2020 Dec 4;15(12):e0242778. doi: 10.1371/journal.pone.0242778 (PMC7717547; doi:10.1371/journal.pone.0242778)
Supplement: Attachment — Submitted filename: Response to the Reviewers3.docx [file pone.0242778.s004.docx]

# Response to the Reviewers

We would like to thank the editor and reviewers for their contributions. Please, find below, in red color, our point by point comments to the reviewers.

Reviewer #2: The present document has been largely improved from its previous version.
In addition, in table1, it’s better to replace the statistical symbol q with Geodetector q.

Author’s response: The authors thank the reviewer for this suggestion. We have replaced the symbol q with Geodetector q in table 1.
